# Association between a combination of cognitively stimulating leisure activities and long-chain polyunsaturated fatty acid intake on cognitive decline among community-dwelling older Japanese individuals

**DOI:** 10.3389/fnagi.2024.1406079

**Published:** 2024-08-07

**Authors:** Hisanori Tokuda, Chika Horikawa, Yukiko Nishita, Yoshihisa Kaneda, Hidenori Obata, Tomohiro Rogi, Takayuki Izumo, Masaaki Nakai, Hiroshi Shimokata, Rei Otsuka

**Affiliations:** ^1^Department of Epidemiology of Aging, National Center for Geriatrics and Gerontology, Ōbu, Aichi, Japan; ^2^Institute for Science of Life, Suntory Wellness Ltd., Soraku-gun, Kyoto, Japan; ^3^Graduate School of Nutritional Sciences, Nagoya University of Arts and Sciences, Nisshin, Aichi, Japan

**Keywords:** cognitive decline, cognitively stimulating leisure activity, long-chain polyunsaturated fatty acid, arachidonic acid, docosahexaenoic acid, eicosapentaenoic acid

## Abstract

Multifactorial lifestyle approaches could be more effective than a single factor for maintaining cognitive function. This study investigated the association of combining cognitively stimulating leisure activities (CSLAs), including puzzles, quizzes, and cognitive training games, with intake of long-chain polyunsaturated fatty acids (LCPUFAs), including docosahexaenoic acid (DHA), eicosapentaenoic acid (EPA), and arachidonic acid (ARA), on cognitive function in the older Japanese individuals without dementia. Participants were community-dwelling Japanese individuals without a self-reported history of dementia (*n* = 906, aged 60–88 years) from datasets of a 2-year longitudinal study (baseline: 2006–2008 and follow-up: 2008–2010). CSLA engagement and LCPUFA intake were divided into high and low groups according to frequency (≥once/week and <once/week) for CSLA engagement and median intake level for LCPUFA intake according to sex, then categorized into four groups. The associations of multivariate-adjusted odds ratio (OR) for a cognitive decline, shown as a decrease in the Mini-Mental State Examination score by 2 or more points, and the combination of CSLA engagement with LCPUFA intake were assessed using a multiple logistic regression model. Subgroup analysis involved participants with low DHA and EPA intakes (*n* = 303; median intake, 325 mg/day), mirroring those in North America. The HIGH-CSLA/HIGH-ARA group cumulatively yielded a lower OR for cognitive decline (0.41; 95% confidence interval, 0.25–0.70) than did the LOW-CSLA/LOW-ARA group (*p* for trend = 0.001). In the subgroup analysis, the OR for the HIGH-CSLA/HIGH-DHA group was notably reduced (0.31; 95% confidence interval, 0.11–0.87; *p* for trend = 0.025) compared with the LOW-CSLA/LOW-DHA group. High CSLA engagement frequency combined with high ARA intake may cumulatively reduce the risk of cognitive decline among older Japanese individuals. Furthermore, combining CSLA engagement frequency with DHA intake could have a positive association with maintaining cognitive function among older individuals, particularly those with low DHA and EPA intakes.

## 1 Introduction

Age-related cognitive decline is a common concern for older people in a super-aged society. The World Health Organization recommends 12 lifestyle changes, including cognitive activities and healthy diets, to reduce cognitive decline risk in older people (World Health Organization, 2019). In general, cognitively stimulating leisure activities (CSLAs) such as crossword or number puzzles and cognitive training games are often used by older individuals with the expectation of maintaining cognitive function. The relationship between cognitive function and CSLA has been studied in older people without dementia. Several interventional studies showed that CSLA, such as word or number puzzles (Jackson et al., 2012; Murphy et al., 2014) and cognitive training games (Nouchi et al., 2012; Hardy et al., 2015) for 4–16 weeks, improved some aspects of cognitive function. In 2-year longitudinal studies, a positive correlation between the frequency of crossword puzzles and *sudoku* engagement and performance of subsequent cognitive function was found in 65-year-old and older European people (*n* = 16,572) (Litwin et al., 2016).

Furthermore, appropriate nutrient intake through a healthy diet also helps maintain cognitive function. Studies have been conducted to understand the relationship between age-related cognitive decline and long-chain polyunsaturated fatty acids (LCPUFAs), primarily from fish, eggs, and meat. LCPUFA, such as docosahexaenoic acid (DHA) and arachidonic acid (ARA), are major components of brain phospholipids. Although their levels in the brain decrease with aging (Söderberg et al., 1991; Carver et al., 2001; McNamara et al., 2008; Freemantle et al., 2012; Tokuda et al., 2014), supplementation with these fatty acids could recover their levels (Tokuda et al., 2014). Several randomized controlled trials (RCTs) have reported the positive effects of DHA, eicosapentaenoic acid (EPA), and ARA supplementation on cognitive function in older individuals without dementia (Ishikura et al., 2009; Yurko-Mauro et al., 2010; Lee et al., 2013; Witte et al., 2014; Tokuda et al., 2015). Furthermore, longitudinal studies have shown that higher DHA, EPA, and ARA intakes were associated with a lower risk for cognitive decline in community-dwelling older Japanese people (Horikawa et al., 2021; Tokuda et al., 2022). The amount of dietary intake of DHA and EPA ranges widely between countries, with the average amount of LCPUFA in Western countries, such as North America, estimated to be at least one-third that of Japanese individuals who have one of the highest DHA and EPA intakes in the world (Kawashima, 2019). Notably, the association between brain health and DHA and EPA intake appeared stronger in populations with lower DHA and EPA intake, akin to that observed in North Americans (Tokuda et al., 2022).

In recent years, multifactorial lifestyle interventions have been focused on as a more effective solution to prevent cognitive decline. The Finnish Geriatric Intervention Study to Prevent Cognitive Impairment and Disability (FINGER) study showed that the 2-year multidomain intervention, including cognitive training and nutritional intervention (e.g., increased fish consumption), improved the global cognitive function in older individuals without dementia (Ngandu et al., 2015). In a longitudinal observational study, a 10-year population-based cohort revealed that a healthy lifestyle partially constructed of cognitive activities (e.g., games at least two times a week) and healthy diets (e.g., daily intake of fish, meat, and eggs) was associated with slower memory decline in cognitively normal older Chinese individuals (Jia et al., 2023). Although these studies show the possibility of combined CSLA with LCPUFA intake being more beneficial to prevent cognitive decline than each activity alone, the association of cognitive decline with a combination of CSLA and LCPUFA has not been studied in older individuals regardless of high or low DHA and EPA intake.

Hence, we hypothesize that high-frequency engagement in CSLA combined with a high intake of LCPUFA may cumulatively reduce the risk of age-related cognitive decline among older Japanese individuals. Furthermore, the subpopulation with DHA and EPA intakes as low as that in the countries with low fish intake, such as North America, could show a stronger positive association of the combination with the preservation of cognitive decline in the Japanese population.

Therefore, the purpose of this study was to evaluate the association of combining CSLA engagement with LCPUFA intake and age-related cognitive decline in older Japanese individuals. The present 2-year longitudinal survey from the National Institute for Longevity Sciences-Longitudinal Study of Aging (NILS-LSA), a Japanese population-based prospective cohort study, was performed to investigate the association of combining CSLA (such as word/number puzzle, quiz, and cognitive training game) engagement with LCPUFA (DHA, EPA, and ARA) intake at baseline and risk of cognitive decline among community-dwelling older Japanese individuals. Additionally, a subgroup analysis based on DHA and EPA intake, comparable to levels in countries with low fish consumption, was conducted within the Japanese population.

## 2 Materials and methods

### 2.1 Study design

The data was obtained from the NILS-LSA, a Japanese population-based prospective cohort study, which included detailed questionnaires, medical check-ups, anthropometric measurements, physical fitness tests, and nutritional examinations to assess the normal aging process. Participants in the first wave (1997–2000) of the NILS-LSA included 2,267 men and women aged between 40 and 79 years (Shimokata et al., 2000). Participants were followed up every 2 years until the seventh wave of the examination, which was completed in 2012; a follow-up survey is still ongoing. Participants included randomly selected, age- and sex-stratified individuals from Obu City and Higashiura Town in the Aichi Prefecture, in cooperation with the local governments. Selected men and women were invited by mail to an explanatory meeting about the examination procedures. Participation was limited to those who understood all examination procedures and provided written informed consent. When participants could not attend the follow-up investigations, the same number of age- and sex-matched random samples were recruited, excluding individuals aged more than 79 years.

### 2.2 Study population

Participants in this 2-year follow-up survey were selected from the sixth (July 2008 to July 2010) and seventh (July 2010 to July 2012) waves of the NILS-LSA by referring to our previous study (Tokuda et al., 2022). The baseline and follow-up data were taken from the sixth and seventh wave surveys, respectively. Of the 2,302 participants aged 40 years or older in the baseline survey, exclusions were based on the following criteria: those who did not participate in the seventh wave (*n* = 315); those whose age was less than or equal to 59 years at the sixth wave because our analysis included only older participants (*n* = 906); those with a self-reported history of dementia at the sixth wave (*n* = 5); those with Mini-Mental State Examination (MMSE) scores less than or equal to 23 at the sixth wave (*n* = 66); those who took EPA drugs (EPADEL^®^) composed of purified EPA ethyl ester (*n* = 11); those with missing data for nutritional assessment variables at the sixth wave (*n* = 65); and those with missing data for the other variables analyzed in this study (*n* = 28). Based on these criteria, longitudinal data from 906 Japanese individuals (460 men and 446 women) aged between 60 and 88 years were available for analysis. The Committee of Ethics of Human Research of the National Center for Geriatrics and Gerontology approved this study (Approval number: 1322), and all the methods were performed in accordance with relevant guidelines and regulations. Written informed consent was obtained from all participants.

### 2.3 Cognitively stimulating leisure activity

The items of the questionnaire for leisure activities in the NILS-LSA were developed by referring to various administrative surveys and a study (Verghese et al., 2003) that surveyed the leisure activities of older adults. Leisure activities including word puzzles (e.g., crosswords), number puzzles (e.g., sudoku), and cognitive training games were categorized as CSLA in the previous study (Ferreira et al., 2015). According to the cited study, leisure activities such as crosswords, sudoku, answering quizzes, and cognitive training games were defined as CSLA in this study. The activities mentioned above were collectively gathered as CSLA when administering the questionnaire survey. CSLA in this study consisted of voluntary activities where individuals seriously attempted to find answers in their daily lives, primarily engaging in these activities alone. The CSLA was implemented as content from newspapers, magazines, TV programs, computers, game consoles, and other similar sources. Research psychometrists assessed the frequency with which each participant engaged in CSLA using a questionnaire survey with the following responses: “never,” “once or several times a year,” “once or several times a month,” “once a week,” “several times a week,” or “every day.” Participants were asked to provide information about their engagement in these activities during the year before study participation.

### 2.4 Nutritional assessment

Nutritional intake per day, including LCPUFA intake and the use of supplements, was assessed using a 3-day dietary record in the sixth wave of the study. The dietary record was completed over 3 continuous days (2 weekdays and 1 weekend day) (Imai et al., 2000). Participants completed the records at home and returned them within a month. The food was either weighed separately on a kitchen scale before cooking, or portion sizes were estimated accordingly. Participants took photographs of their meals before and after eating. The average 3-day food and nutrient intake was calculated according to the Standard Tables of Food Composition in Japan (2010) (Ministry of Education Culture Sports Science and Technology, 2010). Alcohol intake in the previous year was assessed using a food frequency questionnaire. The present study focused on three fatty acids (DHA, EPA, and ARA) as representative LCPUFAs. We created an original supplement database based on the nutritional information of the supplement products used because these supplements often included the LCPUFAs. A new dietary supplement database was developed for the NILS-LSA based on information obtained from the study participants and an additional intensive investigation that was conducted separately (Imai et al., 2006). We asked dietary supplement users to bring these products to the study visit. In addition, when information on the nutrient content was not available to users, we tried to obtain it directly from the manufacturer or distributor of the products. We created a database of dietary supplements that included the names of products, manufacturers, distributors, and nutrient contents in standardized units, such as tablets or capsules. Finally, a database of 902 dietary supplements was constructed. In the actual examination, a self-administered questionnaire, in which we asked whether any dietary supplements had been taken in the previous year, was mailed to the participants. They were asked to record the data themselves at home before the study examination. During the examination, trained dietitians reviewed the questionnaire through an interview that took approximately 10 min. If they had taken any dietary supplements, the name of the product, manufacturer or distributor, serving size and frequency of intake in the previous year were recorded. Nutritional supplements are defined as dietary supplements containing nutritional ingredients in non-natural food forms such as capsules, tablets, powders, and liquids. Energy and nutrient intakes from dietary supplements among “regular users” were estimated using the frequency, amount of intake, and nutrient content in the dietary supplement database. The amounts of LCPUFAs were calculated by adding the values from the dietary records and supplement products, including DHA, EPA, and ARA.

### 2.5 Assessment of cognitive decline

Cognitive function was assessed using the Japanese version of the MMSE (Folstein et al., 1975; Mori et al., 1985). Trained psychologists and researchers administered the test to each wave. The MMSE score ranges from 0 to 30, with higher scores indicating better cognitive function. To estimate a cognitive decline in the present study, a decrease in MMSE score by at least 2 (≤−2) points from baseline was regarded as age-related cognitive decline according to our previous study (Wang et al., 2014; Tokuda et al., 2022). This was in line with previous reports stating that a change in MMSE score by at least 2 points indicated a reliable change in longitudinal repeated assessments in older individuals (Hensel et al., 2007).

### 2.6 Other measurements

Both weight and height were measured on the examination day to calculate the body mass index (BMI, kg/m^2^). Trained interviewers used a questionnaire to assess physical activity regarding the intensity and frequency of activity over the preceding year (Kozakai et al., 2012). The mean amount of total physical activity per day was thus calculated [metabolic equivalents (MET), METs hour/day). A self-completed questionnaire, which was administered approximately 2 weeks before the examination day, was used to collect information on the participant’s history of hypertension (yes/no), dyslipidemia (yes/no), ischemic heart disease (yes/no), stroke (yes/no), diabetes (yes/no), education (≤9, 10–12, or ≥13 years of school), income (≥5.5 or <5.5 million yen/year), and smoking status (yes/no). Depressive tendency (yes/no; ≥16/<15) was assessed using the Japanese version of the CES-D questionnaire (Radloff, 1977; Shima, 1985).

### 2.7 Statistical analyses

All analyses were conducted with the Statistical Analysis System version 9.3 software (SAS Institute, Cary, NC, USA) for the period between 2022 and 2023. Baseline characteristics are expressed as means ± standard deviations (SDs) or median (interquartile range). Differences in baseline characteristics, frequency of CSLA, and LCPUFA intake among participants with and without cognitive decline were assessed the Chi-squared test for categorical variables and either Student’s *t*-test or the Wilcoxon rank-sum test for continuous variables. The main analysis estimated the association between the combination of CSLA engagement frequency with LCPUFA intake at baseline and cognitive decline. Prior to the combination assessment, the association between cognitive decline and CSLA engagement frequency or LCPUFA (DHA, EPA, and ARA) intake was evaluated separately. A decrease in the MMSE score by at least 2 points from the baseline was used as the objective variable. In terms of explanatory variables, CSLA engagement frequency was classified into three groups as follows: (1) high (≥2 times/week), “every day” and “several times a week”; (2) middle (≥once/month), “once a week” and “once or several times a month”; and (3) low (<once/month), “once or several times a year” and “never.” The high and middle group were compared with the low group as the reference. Furthermore, the LCPUFA intake at baseline was divided into tertiles according to sex and compared with the lowest tertile category as the reference. The multiple logistic regression model was used to analyze the odds ratio (OR) and 95% confidence interval (CI) for cognitive decline. The trend associations were assessed by entering dummy variables (–1, 0, 1) assigned to the three groups of CSLA engagement frequency or the tertiles of LCPUFA intake. In model 1, we adjusted for age, sex, education, medical history of hypertension, dyslipidemia, ischemic heart disease, stroke, and diabetes at baseline. Model 2 was further adjusted for confounding variables, including BMI, income, smoking status, alcohol consumption, physical activity, depressive tendency, and MMSE score at baseline. As in the previous study (Tokuda et al., 2022), sex and age were considered basic adjustment factors. Other adjustment factors that had been evaluated at baseline and were reported to be associated with cognitive function and risk of developing dementia were also selected (Livingston et al., 2020). For the combination analysis, we investigated whether CSLA engagement and LCPUFA intake had a cumulative association with the OR for cognitive decline. The combination analysis was performed only if the CSLA engagement frequency or LCPUFA intake each had a positive association with cognitive decline. We established four groups based on the combination of each CSLA group (HIGH or LOW) and LCPUFA intake group (HIGH or LOW). CSLA was divided into two groups according to the frequency of participation: “at least once a week” (HIGH) and “once or several times a month or less” (LOW) (Kurita et al., 2019). On the other hand, we established HIGH and LOW LCPUFA groups based on the median LCPUFA intake according to sex. The multiple logistic regression model was used to analyze the OR for cognitive decline and 95% CI for the three combinations groups (the HIGH-CSLA/HIGH-LCPUFA, HIGH-CSLA/LOW-LCPUFA, and LOW-CSLA/HIGH-LCPUFA groups) compared with the lower frequencies and intakes group (the LOW-CSLA/LOW-LCPUFA). To evaluate the cumulative association, the trend associations were assessed by entering dummy variables (–1, 0, 0, 1) assigned to the four groups. The analysis in models 1 and 2 was also performed. Additionally, we conducted a subgroup analysis focusing on DHA and EPA intakes comparable to those in Western countries, including North America. The average DHA and EPA intake in countries with low fish consumption is estimated to be at least one-third that of the Japanese (Kawashima, 2019). Therefore, participants with the bottom third DHA and EPA intake (*n* = 303) were set as the subgroup with low DHA and EPA intake. According to the analysis for the participants (*n* = 906), the LCPUFA intake at baseline was also divided into tertiles according to sex and compared with the lowest tertile category as the reference in the subgroup. For the combination analysis for CSLA and LCPUFA, DHA or EPA intake was divided into two groups (HIGH^sub^ or LOW^sub^) based on each median DHA or EPA intake according to sex. Subsequent analyses were performed in the same manner as the analysis for the total participants described above. Two-sided *p* values >0.05 were regarded as statistically significant.

## 3 Results

### 3.1 Baseline characteristics of the participants

Table 1 shows the baseline characteristics of all participants and of those with or without a cognitive decline after 2 years. The mean age and percentage of men among the study population (*n* = 906) were 70.2 years and 50.8%, respectively. Approximately half of the participants did not engage in CSLA. The rates of CSLA engagement frequency according to the three groups were 16%, 31%, and 53% for the high, middle, and low groups, respectively. The baseline LCPUFA intakes (mean ± SD) according to the low, middle, and high tertiles were: 101 ± 25, 153 ± 19, and 222 ± 49 mg/day for ARA, respectively; 103 ± 57, 286 ± 59, and 614 ± 252 mg/day for EPA, respectively; and 217 ± 100, 523 ± 98, and 1,041 ± 360 mg/day for DHA, respectively (Table 2). Cognitive decline after 2 years was observed in 180 (19.9%) participants. There were significant differences in age, education, baseline MMSE score, and ARA intake between participants with and without a cognitive decline (Table 1).

**TABLE 1:** Baseline characteristics of the participants (*n* = 906).

	All	Non-cognitive decline	Cognitive decline	*p*
	*n* = 906	*n* = 726	*n* = 180	
Age (years)	70.2 ± 6.7	69.9 ± 6.5	71.2 ± 7.3	0.023
Sex (men %)	50.8 (460)	50.4(366)	52.2 (94)	0.664
BMI (kg/m^2^)	22.7 ± 2.8	22.6 ± 2.8	22.9 ± 2.8	0.232
Education (% ≤9/10–12 / ≥13 years)	26.8/42.8/30.4	23.8/44.6/31.5	38.9/35.6/25.6	<0.001
Alcohol intake (ml/day)	13.0 ± 23.6	13.4 ± 23.6	11.3 ± 23.4	0.274
Smoking status (current %)	8.7 (79)	9.0 (65)	7.8 (14)	0.617
Total physical activity (METs h/day)	34.2 ± 3.0	34.2 ± 2.9	34.3 ± 3.6	0.404
Income (% >5.5 million yen)	37.9 (343)	38.3 (278)	36.1 (65)	0.589
Stroke (%)	5.3 (48)	5.2 (38)	5.6 (10)	0.863
Heart disease (%)	6.8 (62)	6.3 (46)	8.9 (16)	0.225
Hypertension (%)	40.5 (367)	40.4 (293)	41.1 (74)	0.854
Dyslipidemia (%)	26.2 (237)	25.3 (184)	29.4 (53)	0.263
Diabetes (%)	10.4 (94)	10.9 (79)	8.3 (15)	0.316
MMSE	28.1 ± 1.6	27.9 ± 1.6	28.8 ± 1.3	<0001
Depressive tendency (%)	11.1 (101)	10.9 (79)	12.2 (22)	0.609
CSLA (% everyday/STs a week/once a week/once or STs a month/once or STs a year/never)	5.1/10.5/20.2/11.3/7.1/45.9	5.6/10.9/20.8/11.2/6.5/45.0	2.8/8.9/17.8/11.7/9.4/49.4	0.308
ARA (mg/day)	152 (118–190)	154 (120–191)	140 (109–180)	0.005
EPA (mg/day)	280 (150–449)	280 (154–451)	282 (145–447)	0.856
DHA (mg/day)	514 (295–798)	511 (299–798)	545 (270–803)	0.850

Data is presented as means ± SDs or median (interquartile range). Differences in baseline characteristics among participants with non-cognitive decline and cognitive decline were assessed using the Chi-squared test for categorical variables and either Student’s *t*-test or the Wilcoxon rank-sum test for continuous variables. BMI, body mass index; MMSE, Mini-Mental State Examination; CSLA, cognitively stimulating leisure activity; STs, several times; ARA, arachidonic acid; EPA, eicosapentaenoic acid; DHA, docosahexaenoic acid.

**TABLE 2:** Association between ORs for CD in 2 years and CSLA frequency or LCPUFA intakes.

	Model 1			Model 2		
	Low	Middle	High	Low	Middle	High
**CSLA**						
*n*	480	285	141	480	285	141
*n*, CD/NCD	106/374	53/232	21/120	106/374	53/232	21/120
OR	1.000	0.820	0.616	1.000	0.749	0.539
95% CI		0.561–1.198	0.366–1.037		0.504–1.114	0.313–0.927
*p* for trend			0.068			0.025
**LCPUFA intake**						
*n*	301	302	303	301	302	303
**ARA**						
mg/day	101 ± 25	153 ± 19	222 ± 49	101 ± 25	153 ± 19	222 ± 49
*n*, CD/NCD	75/226	58/244	47/256	75/226	58/244	47/256
OR	1.000	0.689	0.577	1.000	0.659	0.544
95% CI		0.464–1.024	0.381–0.873		0.434–1.002	0.352–0.840
*p* for trend			0.009			0.006
**EPA**						
mg/day	103 ± 57	286 ± 59	614 ± 252	103 ± 57	286 ± 59	614 ± 252
*n*, CD/NCD	64/237	53/249	63/240	64/237	53/249	63/240
OR	1.000	0.805	1.014	1.000	0.741	1.038
95% CI		0.532–1.216	0.680–1.510		0.480–1.144	0.683–1.578
*p* for trend			0.947			0.860
**DHA**						
mg/day	217 ± 100	523 ± 98	1,041 ± 360	217 ± 100	523 ± 98	1,041 ± 360
*n*, CD/NCD	62/239	58/244	60/243	62/239	58/244	60/243
OR	1.000	0.949	0.989	1.000	0.864	0.983
95% CI		0.631–1.428	0.660–1.481		0.562–1.327	0.644–1.500
*p* for trend			0.956			0.935

CSLA engagement frequency was classified into three groups: high (≥2 times/week), “every day” and “several times a week”; middle (≥once/month), “once a week” and “once or several times a month”; and low (<once/month), “once or several times a year” and “never.” The baseline LCPUFA intake according to tertiles (low, middle, and high) are shown as means ± SDs. In the analysis, a multiple logistic regression model was adjusted using two models. Model 1: age, sex, education, and medical history (stroke, heart disease, hypertension, dyslipidemia, and diabetes); model 2: model 1 + body mass index, smoking status, alcohol consumption, physical activity, income, depressive tendency, and baseline Mini-Mental State Examination. *n*, number of participants; CD, cognitive decline; NCD, non-cognitive decline; CSLA, cognitively stimulating leisure activity; ARA, arachidonic acid; EPA, eicosapentaenoic acid; DHA, docosahexaenoic acid; OR, odds ratio; CI, confidence interval.

### 3.2 The OR for age-related cognitive decline and CSLA engagement frequency or LCPUFA intake at baseline

Table 2 shows the relationship between the OR for cognitive decline in 2 years and the CSLA engagement frequency or LCPUFA intake at baseline. After multivariable adjustment (model 2), the ORs among the low (<once/month), middle (≥once/month), and high (≥2 times/week) groups of CSLA were 1.000 (reference), 0.749 (95% CI: 0.504–1.114), and 0.539 (95% CI: 0.313–0.927), respectively. The significant trend association among the three groups in CSLA engagement (*p* for trend = 0.025) showed that higher CSLA engagement frequency was associated with a lower OR for cognitive decline. Regarding LCPUFA intake, the ORs for the low, middle, and high tertiles of ARA intake were 1.000 (reference), 0.659 (95% CI: 0.434–1.002), and 0.544 (95% CI: 0.352–0.840), respectively. The significant trend association assigned to the tertile of ARA intake (*p* for trend = 0.006) showed that higher ARA intake was associated with a lower OR for cognitive decline. No significant relationship was observed between the OR and DHA or EPA intake.

### 3.3 The OR for age-related cognitive decline and the combination of CSLA engagement with LCPUFA intake at baseline

**FIGURE 1:**
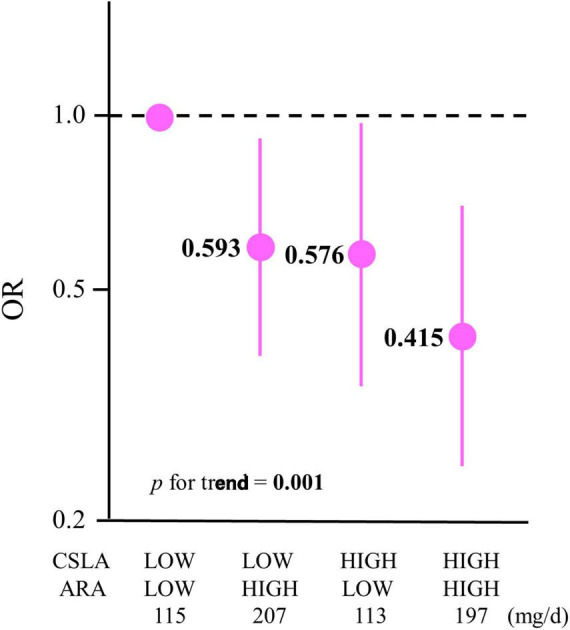
Association between ORs for cognitive decline in 2 years and a combination of CSLA engagement and ARA intake. The participants were divided into four groups based on a combination of CSLA frequency (HIGH: ≥once/week and LOW: <once/week) and ARA intake (HIGH: ≥median and LOW: <median): LOW-CSLA/LOW-ARA (*n* = 302), LOW-CSLA/HIGH-ARA (*n* = 280), HIGH-CSLA/LOW-ARA (*n* = 151), and HIGH-CSLA/HIGH-ARA (*n* = 173). Data are expressed as ORs and 95% confidence intervals. ORs are shown in a log scale. The baseline mean ARA intakes (mg/day) among the groups are shown. Multiple logistic regression model adjusted by model 2: age, sex, education, medical history (stroke, heart disease, hypertension, dyslipidemia, and diabetes), body mass index, smoking status, alcohol consumption, physical activity, income, depressive tendency, and baseline Mini-Mental State Examination. CSLA, cognitively stimulating leisure activity; ARA, arachidonic acid; OR, odds ratio.

The combination analysis was performed only if the CSLA engagement frequency or LCPUFA intake each had a frequency- or dose-dependent association with cognitive decline, respectively. The exposures for the combination were the CSLA engagement frequency and ARA intake at baseline because these were inversely associated with the OR for cognitive decline. The participants were divided into four groups based on the combination of CSLA frequency (HIGH and LOW) and ARA intake (HIGH and LOW). Divided into two groups for CSLA, the participants in the HIGH-CSLA (≥once/week) group and those in the LOW-CSLA (<once/week) group were 36% and 64% in all participants, respectively. Moreover, baseline ARA intake (means ± SD) divided into two groups based on median by sex were 114 ± 29 mg/day in the LOW-ARA group and 203 ± 49 mg/day in the HIGH-ARA group. After multivariable adjustment (model 2), the ORs for cognitive decline among each group with the LOW-CSLA/LOW-ARA group as a reference are shown in Figure 1 and Supplementary Table 1. The OR for the HIGH-CSLA/HIGH-ARA group was 58.5% lower compared to the reference group. The significant trend association among the four groups (*p* for trend = 0.001) showed that the decrease rate for the OR in the HIGH-CSLA/HIGH-ARA group was cumulatively larger than that in the HIGH-CSLA/LOW-ARA (42.4%) or LOW-CSLA/HIGH-ARA groups (40.7%).

### 3.4 The subgroup analysis by low DHA and EPA intakes

The subgroup analysis based on DHA and EPA intake as low as that in the countries with low fish intake was performed, and the baseline characteristics in the subgroup (*n* = 303) are shown in Supplementary Table 2. Mean intakes of DHA and EPA (220 and 105 mg/day, respectively) in the subgroup participants were one-third of those in all participants (594 and 335 mg/day, respectively). In addition, the baseline DHA and EPA intakes (mean ± SD) according to low, middle, and high tertiles were: 106 ± 41, 221 ± 43, and 330 ± 56 mg/day for DHA, respectively, and 38 ± 21, 105 ± 24, and 172 ± 32 mg/day for EPA, respectively. Supplementary Table 3 shows that a higher DHA intake was associated with a lower OR for cognitive decline (*p* for trend = 0.023). However, no significant association between EPA intake and cognitive decline was observed.

**FIGURE 2:**
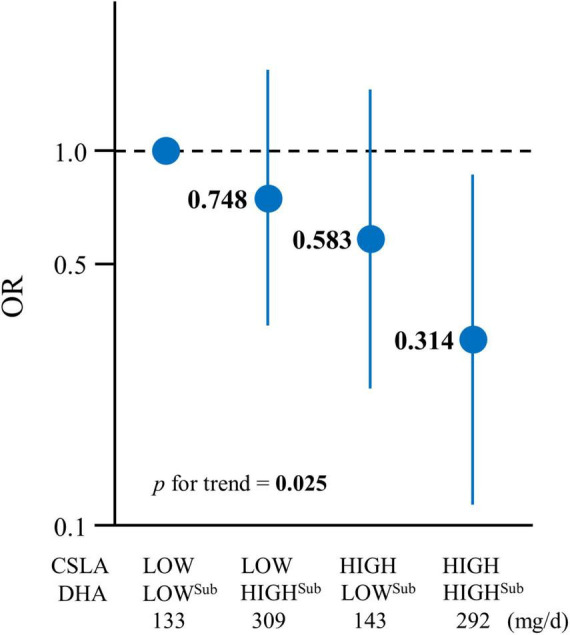
Association between cognitive decline and CSLA and DHA intake in the low DHA + EPA intake subgroup. The participants were divided into four groups based on a combination of CSLA frequency (HIGH: ≥once/week and LOW: <once/week) and DHA intake (HIGH^sub^: ≥median and LOW^sub^: <median): LOW-CSLA/LOW^sub^-DHA (*n* = 94), LOW-CSLA/HIGH^sub^-DHA (*n* = 96), HIGH-CSLA/LOW^sub^-DHA (*n* = 57), and HIGH-CSLA/HIGH^sub^-DHA (*n* = 56). Data are expressed as ORs and 95% confidence intervals. ORs are shown in a log scale. The baseline mean DHA intakes (mg/day) among the groups are shown. Multiple logistic regression model adjusted by model 2: age, sex, education, medical history (stroke, heart disease, hypertension, dyslipidemia, and diabetes), body mass index, smoking status, alcohol consumption, physical activity, income, depressive tendency, and baseline Mini-Mental State Examination. CSLA, cognitively stimulating leisure activity; DHA, docosahexaenoic acid; EPA, eicosapentaenoic acid; OR, odds ratio.

To evaluate the association between a cognitive decline and the combination of the CSLA engagement and DHA intake, the participants in the subgroup analysis were divided into four groups based on the combination of CSLA frequency (HIGH and LOW) and DHA intake (HIGH^sub^ and LOW^sub^). The baseline DHA intake (mean ± SD) in the subgroup analysis, divided into two groups based on the median by sex, was 137 ± 57 mg/day in the LOW*^sub^*-DHA group and 303 ± 64 mg/day in the HIGH*^sub^*-DHA group. Figure 2 and Supplementary Table 4 show the multivariable-adjusted OR for cognitive decline among each group with the LOW-CSLA/LOW-DHA^sub^ group as a reference. The OR in the HIGH-CSLA/HIGH-DHA^sub^ group was 68.6% lower compared to the reference group (model 2). The significant trend association among the four groups (model 2, *p* for trend = 0.025) showed that the HIGH-CSLA/HIGH-DHA^sub^ group had a cumulatively larger decrease rate for the OR compared to the HIGH-CSLA/LOW-DHA^sub^ (41.7%) or LOW-CSLA/HIGH-DHA^sub^ groups (25.2%).

## 4 Discussion

In this 2-year longitudinal study, we assessed the combined impact of CSLA—including word and number puzzles, quizzes, and cognitive training games—with LCPUFA intake (DHA, EPA, and ARA) on cognitive decline among community-dwelling older Japanese individuals (*n* = 906). This study showed that higher CSLA engagement frequency and higher ARA, but not DHA or EPA, intake were associated with lower risks of cognitive decline. Furthermore, it was found that the combination of high CSLA engagement with high ARA intake cumulatively yielded a lower risk for cognitive decline. In the subgroup analysis, higher DHA intake corresponded to a reduced risk of cognitive decline among participants (*n* = 303) with DHA and EPA intakes comparable to those in typical Western countries. In addition, the combination of high CSLA engagement frequency with high DHA intake was found to cumulatively lower the risk of cognitive decline.

Participants in our cohort study represented a population with a typical frequency of CSLA engagement and higher DHA and EPA intakes compared to other countries. In studies that identified crossword or sudoku puzzles as representative of CSLA, the proportion of participants engaging in CSLA at least once a week in this study (36%) aligned with that of 5,300 older Japanese individuals (34%) and 19,078 older UK individuals (37%) (Brooker et al., 2019a; Kurita et al., 2019). This suggests that the older Japanese individuals in the present study could be regarded as an older population with a general frequency of CSLA engagement. Regarding LCPUFA intake, participants in this study can be considered a population with higher DHA and EPA intake compared to those in other countries. The mean intake of DHA (594 mg/day) and EPA (335 mg/day) in this study was observed to be quite larger than that in countries with low fish intake, such as North America (DHA, 60–190 mg/day; EPA, 20–50 mg/day), while it aligned to that of countries with high DHA and EPA intake such as Finland (DHA, 420–510 mg/day; EPA, 160–190 mg/day) (Kawashima, 2019). Therefore, we conducted a subgroup analysis based on DHA and EPA intakes that were as low as those in countries with low fish intakes. The mean intake of DHA and EPA in the subgroup population was 220 and 105 mg/day, respectively, which was comparable to that in the North American population (Kawashima, 2019). This consistency suggests that the subgroup population was matched to the target group in the present study.

As for the relationship between CSLA engagement or LCPUFA intake and maintaining cognitive function, the results in the 2-year longitudinal study aligned with those from previous studies. Several RCTs showed that CSLAs, such as word or number puzzles and cognitive training games, 5–7 days/week for 4–16 weeks, improved some cognitive domains (Jackson et al., 2012; Nouchi et al., 2012; Murphy et al., 2014; Hardy et al., 2015). Moreover, longitudinal and cross-sectional studies reported that CSLAs once a month to every day had positive associations with cognitive functions (Litwin et al., 2016; Brooker et al., 2019a,b; Kurita et al., 2019). Our findings showed that CSLA engagement at least twice a week reduced the risk of cognitive decline by 46% compared with less than once a month. Although the validity of the frequency of CSLA in this study could be supported by a range of previous studies, the suitable frequency and implementation time per session remain unclear. Further studies are needed to evaluate minimum levels of CSLA engagement in maintaining cognitive function in older individuals without dementia.

In terms of LCPUFA intake, this study showed that ARA intake exhibited an inverse association with the risk of cognitive decline in community-dwelling older Japanese individuals. This finding could be supported by several RCTs and longitudinal observational studies in countries with high DHA and EPA intake. In fact, the efficacy of ARA supplementation on cognitive function has been reported in RCTs in older Japanese individuals without dementia (Ishikura et al., 2009; Tokuda et al., 2015). Furthermore, it has been reported that ARA intake at baseline was inversely associated with the risk of cognitive decline after 2–4 years in community-dwelling older Japanese individuals (Horikawa et al., 2021; Tokuda et al., 2022). In terms of dietary sources of ARA, animal products such as eggs, fish, and meat include ARA. Japanese people have a balanced intake of ARA from a variety of animal products (Kawashima, 2019). The previous study showed that the sources of ARA intake were egg (ca. 30%), fish (ca. 30%–40%), and meat (ca. 25%) in older Japanese people (Kawabata et al., 2011). In contrast, the present longitudinal study found no significant association between the risk of cognitive decline and DHA or EPA intake in the same participants. Previous RCTs and longitudinal observational studies have shown that DHA and EPA intake had a protective association with cognitive decline in older individuals (Yurko-Mauro et al., 2010; Lee et al., 2013; Daiello et al., 2015; Wei et al., 2023). However, these studies were mainly conducted in countries with low fish intake (such as United States, Netherlands, France, and Malaysia). The discrepancy might be attributed to the variance in baseline DHA and EPA intakes between countries with low and high fish consumption. It appears that the protective correlation of DHA and EPA intake against cognitive decline is more pronounced in populations with insufficient DHA and EPA levels compared to those with adequate intakes. This hypothesis gains partial support from the subgroup analysis conducted in this study, where a higher DHA intake correlated with a reduced risk of cognitive decline among individuals with low DHA and EPA levels, akin to those observed in North American populations. However, an inverse association of cognitive decline with DHA intake was also reported in community-dwelling older Japanese individuals without any range restriction of DHA and EPA intake (Horikawa et al., 2021). Taken together, DHA intake may reduce the risk of cognitive decline in older individuals without dementia and with low fish intake more than in those with high fish intake. Furthermore, it was suggested that the association between cognitive decline and the intake of DHA should be carefully evaluated, especially in a population with high DHA and EPA intakes.

Recently, the FINGER study has heightened focus on multifactorial approaches to maintain cognitive function (Ngandu et al., 2015). To the best of our knowledge, this is the first study to investigate the combination of CSLA engagement and LCPUFA intake on cognitive function in older individuals without dementia. This study found that the combination of high CSLA engagement (≥1/week) with high ARA intake (ca. 200 mg/day) yielded a 59% risk reduction of cognitive decline compared with that of low CSLA engagement (<1/week) with low ARA intake (ca. 110 mg/day). Furthermore, the trend association analysis showed that the risk reduction of cognitive decline in high CSLA engagement with high ARA intake (59%) individuals was larger than that in high CSLA engagement (42%) or high ARA intake (41%) individuals alone. This suggests that the combination of high CSLA engagement frequency and high ARA intake could cumulatively contribute to maintaining cognitive function in older individuals without dementia. Regarding CSLA engagement and DHA intake, it was observed that the combination of high CSLA engagement frequency with high DHA intake cumulatively reduced the risk of cognitive decline in the population with low DHA and EPA intakes. This finding was partially supported by the previous study in China, where DHA and EPA intakes are lower than those in Japan (Kawashima, 2019). The COAST study reported that a healthy lifestyle, partially constructed of CSLA (games at least twice/week) and healthy diets (daily intake of fish, meat, and eggs), was associated with slower memory decline for 10 years in cognitively normal older Chinese individuals (Jia et al., 2023). These studies suggest that the combination of high CSLA engagement with appropriate LCPUFA (DHA and ARA) intake could be more beneficial in preventing age-related cognitive decline than CSLA engagement or LCPUFA intake alone in older individuals. Further studies are required to clarify this possibility.

In terms of the mechanism of the combination of high CSLA engagement and high LCPUFA (DHA and ARA) intake on a lower risk of cognitive decline, no promising hypotheses have been presented. Although cellular and molecular mechanisms are difficult to explain, it may be plausible that CSLAs and LCPUFAs contribute to the same cognitive domain; therefore, their combination strengthens their individual effect on reducing the risk of cognitive decline. Several interventional studies showed that CSLAs improved executive function, processing speed, and working memory (Jackson et al., 2012; Nouchi et al., 2012; Murphy et al., 2014; Hardy et al., 2015). As for LCPUFAs, the efficacy of supplementation of DHA and ARA on attention and working memory in older individuals without dementia has also been reported (Ishikura et al., 2009; Lee et al., 2013; Tokuda et al., 2015). The prefrontal cortex is a well-known region associated with executive function, attention, and working memory (Criaud and Boulinguez, 2013; Rabinovici et al., 2015). Based on the results of the previously mentioned intervention studies, it is possible that CSLAs and LCPUFA intake cooperated to maintain prefrontal cortex function and may have been involved in reducing the risk of global cognitive decline. Another potential mechanism is that the combination of functional and organic approaches may have a cumulative effect on cognitive function in older individuals. Additionally, the brain’s DHA and ARA decrease with age (Söderberg et al., 1991; Carver et al., 2001; McNamara et al., 2008; Freemantle et al., 2012; Tokuda et al., 2014), and supplementation of these LCPUFAs could recover their levels (Tokuda et al., 2014). This suggests a possibility that adding a functional approach of cognitive stimulation to the organic approach of supplementing DHA and ARA, which decrease in the brain with aging, may cooperatively produce additive effects in attenuating age-related cognitive decline. Aside from the combination analysis, the present study did not find that EPA had a protective association with cognitive decline in older Japanese individuals without dementia, regardless of the population with high or low DHA and EPA intake. This finding may be understandable as major LCPUFAs in brain phospholipids are constructed with DHA and ARA, not EPA (Chen and Bazinet, 2015). In fact, DHA, but not EPA supplementation, improved executive functions in older individuals with mild cognitive impairment (Sinn et al., 2012). However, further studies are needed to clarify the strength of the contribution of DHA or EPA intake on maintaining cognitive function in older individuals without dementia.

The present study had strengths. To the best of our knowledge, this is the first study to report a positive association between the risk of cognitive decline and the combination of CSLA engagement with LCPUFA intake in older individuals. Next, the amount of LCPUFA intake at baseline reflected real-life values because it was calculated not only from the daily diet but also from supplements. However, this study also had several limitations. First, CSLA cannot be categorized into activities such as crosswords, quizzes, and cognitive training games because relevant activities were collected together as CSLA when questionnaires were used. Second, the CSLA in this study may somewhat differ from the cognitive training game designed for improving cognitive function in the previous RCTs. The CSLA included crosswords, sudoku, answering quizzes, and cognitive training games, which were primarily recreational activities, not specifically designed to improve cognitive function. Careful consideration may be needed when comparing these activities. Furthermore, the engagement times of CSLA in a session cannot be shown because of a lack of available data although the load on cognitive activity time per session would be quite important to affect cognitive decline. A standard engagement time per session and frequency in a week would be important information for social implementation. Further studies are necessary to set a standard of CSLA for reducing the risk of age-related cognitive decline. Third, a 2-year follow-up period may not be an adequate interval to observe the influences of exposure on cognitive decline. Fourth, the MMSE is a screening tool for dementia and not a diagnostic tool. Cognitive decline was evaluated only by the MMSE. Although the MMSE is widely used to assess global cognitive function, this study was unable to evaluate the association between specific cognitive domains and CSLA or LCPUFA intake. Fifth, the frequency of CSLA engagement and LCPUFA intake were obtained from one baseline assessment. Therefore, CSLA frequency and habitual daily intake may change during the follow-up period. Sixth, the association between LCPUFA levels in blood at baseline and the risk of cognitive decline was not evaluated because there was no data on blood LCPUFA levels. However, it was assumed that there is a positive correlation between blood and intake levels. A positive correlation between the dietary intake of DHA and EPA and their blood levels has, in fact, been reported (Hodson et al., 2008). Interventional studies have shown a dose-dependent correlation between ARA intake and changes in blood ARA levels (Kawashima, 2019). Further studies on the association between cognitive decline and blood LCPUFA levels are required. Finally, the findings in this study are based on the data from participants who could be followed up for 2 years. The participants lost to follow-up were older, had lower education levels, a higher prevalence of diabetes, lower MMSE scores, and higher depressive tendency (data not shown). It is uncertain whether the results would remain consistent if the lost to follow-up group were included. Given these limitations, intervention studies are needed to confirm whether the combination of CSLA as a leisure activity and LCPUFA intake improves cognitive function in older adults for the next step.

## 5 Conclusion

In conclusion, a high CSLA engagement frequency combined with a high ARA intake was associated with a cumulatively reduced risk of cognitive decline among older Japanese individuals. Furthermore, a combination of high CSLA engagement frequency with a high DHA intake could have a positive association with cumulatively maintaining cognitive function among older individuals with low DHA and EPA intakes, such as those seen in countries with low fish intake. Hence, combining high CSLA engagement with appropriate intake of LCPUFAs (DHA and ARA) may be more beneficial for preventing age-related cognitive decline in older individuals than either CSLA engagement or LCPUFA intake alone. Further interventional studies are required to clarify the findings of this study.

## Data availability statement

The original contributions presented in this study are included in this article/Supplementary material. The datasets generated and analyses performed as part of the current study are not publicly available due to the consent requirements of the participants. However, participant characteristics, including sex and age, stratified descriptive data, are available from the corresponding author upon reasonable request.

## Ethics statement

The studies involving humans were approved by the Committee of Ethics of Human Research of the National Center for Geriatrics and Gerontology (Approval number: 1322). The studies were conducted in accordance with the local legislation and institutional requirements. The participants provided their written informed consent to participate in this study.

## Author contributions

HT: Conceptualization, Formal analysis, Writing – original draft, Writing – review & editing. CH: Conceptualization, Writing – review & editing. YN: Data curation, Supervision, Writing – review & editing. YK: Project administration, Supervision, Writing – review & editing. HO: Project administration, Supervision, Writing – review & editing. TR: Supervision, Writing – review & editing. TI: Project administration, Supervision, Writing – review & editing. MN: Project administration, Supervision, Writing – review & editing. HS: Conceptualization, Data curation, Supervision, Writing – review & editing. RO: Conceptualization, Data curation, Funding acquisition, Supervision, Writing – review & editing.
