# Supplementary material for: Association between a combination of cognitively stimulating leisure activities and long-chain polyunsaturated fatty acid intake on cognitive decline among community-dwelling older Japanese individuals
Source: Front Aging Neurosci. 2024 Aug 7;16:1406079. doi: 10.3389/fnagi.2024.1406079 (PMC11335671; doi:10.3389/fnagi.2024.1406079)
Supplement: Supplementary file 1 [file Table_1.pdf]

Supplementary Material

Supplementary Table 1. Association between OR for CD in 2 years and the combination of CSLA engagement and ARA intake.

	Model 1				Model 2			
**CSLA**	LOW	LOW	HIGH	HIGH	LOW	LOW	HIGH	HIGH
**ARA**	LOW	HIGH	LOW	HIGH	LOW	HIGH	LOW	HIGH
ARA intake (mg/d)	115 ± 29	207 ± 49	113 ± 29	197 ± 49	115 ± 29	207 ± 49	113 ± 29	197 ± 49
n	302	280	151	173	302	280	151	173
n, CD/NCD	81/221	46/234	27/124	26/147	81/221	46/234	27/124	26/147
OR	1.000	0.550	0.575	0.487	1.000	0.593	0.576	0.415
95% CI		0.364–0.832	0.347–0.952	0.295–0.803		0.383–0.918	0.340–0.976	0.246–0.699
p for trend				0.005				0.001

The participants were divided into four groups based on the combination of CSLA frequency (HIGH: ≥once/week and LOW: <once/week) and ARA intake (HIGH: ≥median and LOW: <median): LOW-CSLA/LOW-ARA, LOW-CSLA/HIGH-ARA, HIGH-CSLA/LOW-ARA, and HIGH-CSLA/HIGH-ARA groups. The baseline ARA intakes among groups are shown as means ± standard deviations.

In the analysis, a multiple logistic regression model was adjusted using two models. Model 1: age, sex, education, and medical history (stroke, heart disease, hypertension, dyslipidemia, and diabetes); Model 2: Model 1 + body mass index, smoking status, alcohol consumption, physical activity, income, depressive tendency, and baseline Mini-Mental State Examination.

n, number of participants; CD, cognitive decline: NCD, non-cognitive decline; CSLA, cognitively stimulating leisure activities; ARA, arachidonic acid; OR, odds ratio; CI, confidence interval.

Supplementary Table 2. Baseline characteristics of the participants in the subgroup with low DHA and EPA intakes (n = 303).

		All n=303	Non-Cognitive decline n=242	Cognitive decline n=61	*p*
Age	(years)	70.3 ± 6.8	69.8 ± 6.6	72.2 ± 7.3	0.015
Sex	(Men %)	50.2	53.3	37.7	0.029
BMI	(kg/m^2)	22.6 ± 2.8	22.5 ± 2.8	23.0 ± 2.6	0.200
Education	(% ≤9/10–12/≥13 years)	27.7/41.9/30.4	24.8/41.7/33.5	39.3/42.6/18.0	0.023
Alcohol	(mL/d)	12.3 ± 23.7	13.0 ± 23.9	9.5 ± 22.8	0.311
Smoking status	(Current%)	8.3 (25)	9.1 (22)	4.9 (3)	0.290
Total physical activity	(METs h/d)	34.0 ± 3.5	33.8 ± 2.9	35.0 ± 5.1	0.092
Income	(% >5.5 million yen)	34.7 (105)	34.3 (83)	36.1 (22)	0.795
Stroke	(%)	7.6 (23)	6.6 (16)	11.5 (7)	0.200
Heart disease	(%)	5.6 (17)	5.0 (12)	8.2 (5)	0.326
Hypertension	(%)	40.3 (122)	39.7 (96)	42.6 (26)	0.674
Dyslipidemia	(%)	27.7 (84)	25.2 (61)	37.7 (23)	0.051
Diabetes	(%)	7.9 (24)	8.3 (20)	6.6 (4)	0.659
MMSE		28.1 ± 1.6	27.9 ± 1.6	28.6 ± 1.5	0.001
Depressive tendency	(%)	14.2 (43)	15.7 (38)	8.2 (5)	0.133
CSLA	(% everyday/STs a week/once a week/once or STs a month/once or STs a year/never)	4.6/10.2/22.4/12.9/8.3/41.6	5.4/10.3/23.1/13.2/6.6/41.3	1.6/9.8/19.7/11.5/14.8/42.6	0.327
ARA	(mg/d)	124 (95, 153)	126 (103, 161)	107 (76, 134)	<0.001
EPA	(mg/d)	107 (57, 153)	108 (58, 156)	97 (56, 145)	0.341
DHA	(mg/d)	223 (133, 296)	230 (135, 303)	192 (109, 275)	0.055

Data is presented as means ± standard deviations or median (interquartile range). Differences in baseline characteristics among participants with non-cognitive decline and cognitive decline were assessed using the chi-squared test for categorical variables and either Student's t-test or the Wilcoxon rank-sum test for continuous variables.

BMI, body mass index; MMSE, Mini-Mental State Examination; CSLA, cognitively stimulating leisure activities; STs, several times; ARA, arachidonic acid; EPA, eicosapentaenoic acid; DHA, docosahexaenoic acid.

Supplementary Table 3. Association between ORs for CD in 2 years and EPA or DHA intakes at baseline in the subgroup with low DHA + EPA intake.

		Model 1			Model 2		
		**Low**sub	**Middle**sub	**High**sub	**Low**sub	**Middle**sub	**High**sub
	n	100	101	102	100	101	102
**EPA**	mg/d	38 ± 21	105 ± 24	172 ± 32	38 ± 21	105 ± 24	172 ± 32
	n, CD/NCD	20/80	22/79	19/83	20/80	22/79	19/83
	OR	1.000	1.166	0.980	1.000	1.133	0.803
	95% CI		0.574–2.367	0.471–2.041		0.538–2.387	0.366–1.762
	p for trend			0.958			0.585
**DHA**	mg/d	106 ± 41	221 ± 43	330 ± 56	106 ± 41	221 ± 43	330 ± 56
	n, CD/NCD	23/77	24/77	14/88	23/77	24/77	14/88
	OR	1.000	0.984	0.506	1.000	0.982	0.385
	95% CI		0.497–1.948	0.237–1.083		0.475–2.029	0.169–0.875
	p for trend			0.079			0.023

The baseline LCPUFA intakes according to tertiles (low^{sub}, middle^{sub}, and high^{sub}) are shown as means ± standard deviations.

In the analysis, a multiple logistic regression model was adjusted using two models. Model 1: age, sex, education, and medical history (stroke, heart disease, hypertension, dyslipidemia, and diabetes); Model 2: Model 1 + body mass index, smoking status, alcohol consumption, physical activity, income, depressive tendency, and baseline Mini-Mental State Examination.

n, number of participants; CD, cognitive decline: NCD, non-cognitive decline; EPA, eicosapentaenoic acid; DHA, docosahexaenoic acid; OR, odds ratio; CI, confidence interval.

Supplementary Table 4. Association between ORs for CD in 2 years and the combination of CSLA engagement and DHA intake in the subgroup with low DHA + EPA intake.

	Model 1				Model 2			
**CSLA**	LOW	LOW	HIGH	HIGH	LOW	LOW	HIGH	HIGH
**DHA**	LOW^{sub}	HIGH^{sub}	LOW^{sub}	HIGH^{sub}	LOW^{sub}	HIGH^{sub}	LOW^{sub}	HIGH^{sub}
DHA intake (mg/d)	133 ± 55	309 ± 63	143 ± 61	292 ± 64	133 ± 55	309 ± 63	143 ± 61	292 ± 64
n	94	96	57	56	94	96	57	56
n, CD/NCD	22/72	20/76	10/47	9/47	22/72	20/76	10/47	9/47
OR	1.000	0.840	0.656	0.427	1.000	0.748	0.583	0.314
95% CI		0.406–1.740	0.273–1.579	0.166–1.094		0.341–1.643	0.232–1.462	0.114–0.866
p for trend				0.076				0.025

The participants were divided into four groups based on the combination of CSLA frequency (HIGH: ≥once/week and LOW: <once/week) and DHA intake (HIGH^{sub}: ≥median and LOW^{sub}: <median): LOW-CSLA/LOW^{sub}-DHA, LOW-CSLA/HIGH^{sub}-DHA, HIGH-CSLA/LOW^{sub}-DHA, and HIGH-CSLA/HIGH^{sub}-DHA groups. The baseline DHA intakes among the groups are shown as means ± standard deviations.

In the analysis, a multiple logistic regression model was adjusted using two models. Model 1: age, sex, education, and medical history (stroke, heart disease, hypertension, dyslipidemia, and diabetes); Model 2: Model 1 + body mass index, smoking status, alcohol consumption, physical activity, income, depressive tendency, and baseline Mini-Mental State Examination.

n, number of participants; CD, cognitive decline: NCD, non-cognitive decline; CSLA, cognitively stimulating leisure activities; DHA, docosahexaenoic acid; OR, odds ratio; CI, confidence interval.
